# Oxidative Stress and Its Molecular Effectors in Uterine Smooth Muscle Tumors: Bridging Epigenetic Regulation and Glycoprotein Biomarkers

**DOI:** 10.3390/antiox15101252

**Published:** 2026-09-30

**Authors:** Areti Kourti, Paraskevi Karioti, Foteini Chouliara, Elisavet Georgiou, Kali Makedou, Ioannis Kalogiannidis

**Affiliations:** 1 Laboratory of Biological Chemistry, School of Medicine, Faculty of Health Sciences, Aristotle University of Thessaloniki, GR-54124 Thessaloniki, Greece; elgeorgiou@auth.gr; 2 3rd Department of Obstetrics and Gynecology, Hippokratio General Hospital of Thessaloniki, School of Medicine, Faculty of Health Sciences, Aristotle University of Thessaloniki, GR-54124 Thessaloniki, Greece; kariotiparaskevi@gmail.com (P.K.); foteinichouliara3@gmail.com (F.C.); 3 Laboratory of Biochemistry, AHEPA University Hospital, School of Medicine, Aristotle University of Thessaloniki, GR-54124 Thessaloniki, Greece; kmakedou@auth.gr

**Keywords:** oxidative stress, diagnostic biomarkers, uterine smooth muscle tumors, galectin-1, microRNAs, genomic instability, tumor microenvironment

## Abstract

Background: Uterine smooth muscle tumors present a diverse clinical spectrum, ranging from ubiquitous benign leiomyomas to highly lethal uterine leiomyosarcomas (uLMSs). The preoperative differentiation of these entities, alongside the intermediate smooth muscle tumors of uncertain malignant potential (STUMP), remains an unresolved clinical challenge. Methods: This comprehensive review synthesizes the current literature on the pathogenesis of uterine smooth muscle tumors, focusing on the role of oxidative stress and its downstream molecular effectors. We evaluate the interplay between oxidative stress markers, epigenetic regulation via microRNAs (miRNAs), and circulating glycoproteins. Results: Increased oxidative stress is a hallmark associated with uLMS, characterized by altered antioxidant enzyme activity and elevated lipid peroxidation products such as malondialdehyde (MDA). This oxidative burden is associated with DNA damage and profound genomic instability, participating in the evolution of the malignant phenotype. Concurrently, the stressed tumor microenvironment exhibits significant epigenetic deregulation. Tumor-suppressive miRNAs (e.g., the miR-29 and miR-200 families) are downregulated, while oncogenic miRNAs (e.g., miR-21) are upregulated, promoting survival and proliferation pathways. Furthermore, galectin-1, a glycoprotein responsive to microenvironmental stress, is elevated in the serum of uLMS and STUMP patients, demonstrating high specificity in investigated cohorts, though limited sensitivity (47%), whereas mucin-1 (MUC1) shows no significant diagnostic utility. Conclusions: Oxidative stress is strongly associated with the genomic instability and molecular derangements observed in uterine sarcomas. Integrating oxidative stress indicators with specific miRNA signatures and galectin-1 levels could potentially yield robust, non-invasive diagnostic panels. However, prospective validation in large, independent cohorts is strictly required before these markers can be implemented to improve preoperative risk stratification and guide targeted therapeutic interventions.

## 1. Introduction

Cancers of the female reproductive tract present complex diagnostic and therapeutic hurdles, particularly concerning mesenchymal neoplasms originating in the myometrium. Uterine leiomyomas represent the most frequent benign tumors in women of reproductive age, affecting 15–25% of women clinically and reaching an epidemiological prevalence of up to 70% by age 50 [1,2]. These benign tumors cause significant morbidity, presenting with symptoms that include abnormal uterine bleeding (menorrhagia), severe pelvic pain, dysmenorrhea, pressure on adjacent organs leading to urinary or bowel dysfunction, and significant challenges with fertility [1].

In stark contrast to these benign lesions, uterine sarcomas are exceedingly rare, comprising approximately 3% of all uterine cancers [1,3]. Despite their rarity, these malignancies are highly aggressive, characterized by rapid cellular proliferation, early hematogenous dissemination, and a dismal prognosis. Epidemiological data indicate that uLMS predominantly affects peri- and postmenopausal women, with a median age at diagnosis between 50 and 55 years [4]. The incidence rate of uLMS ranges from a mere 0.36 to 0.64 per 100,000 women annually [4]. Five-year survival rates for uLMS are exceptionally poor, ranging from 18.8% to 65%, with recurrence rates soaring between 53% and 71% even after complete surgical resection. Risk factors for these aggressive sarcomas remain scarcely defined but include hereditary retinoblastoma, Li–Fraumeni syndrome (inherited TP53 mutations), prior pelvic exposure to radiation, and the therapeutic administration of tamoxifen [4,5]. Between these two extremes of benign and malignant disease lies a transitional category known as smooth muscle tumors of uncertain malignant potential (STUMP), which exhibit overlapping histopathological features and an unpredictable clinical course characterized by occasional metastasis [6].

The preoperative differentiation of benign leiomyomas from uLMS and STUMP remains a profound clinical dilemma. Current diagnostic methodologies rely heavily on clinical evaluation and imaging techniques, such as ultrasound and magnetic resonance imaging (MRI), which frequently exhibit substantial overlapping features. Consequently, a definitive diagnosis is predominantly established postoperatively following the meticulous histopathological examination of the surgically excised specimen. This diagnostic ambiguity poses severe clinical risks; the inadvertent use of minimally invasive surgical techniques, specifically laparoscopic power morcellation, can inadvertently disseminate occult malignant tissue throughout the peritoneal cavity, drastically worsening patient outcomes [7].

To address this critical diagnostic gap, contemporary research has pivoted toward the molecular landscape of these tumors. Oxidative stress is highly associated with oncogenesis in the female reproductive tract. The imbalance between reactive oxygen species (ROS) production and cellular antioxidant defense mechanisms may contribute to severe DNA damage and genomic instability, participating in malignant transformation [1]. This manuscript provides a comprehensive review of the pathogenesis of uterine smooth muscle tumors, investigating oxidative stress as a major factor associated with genomic instability, epigenetic dysregulation via miRNAs, and the expression of circulating glycoprotein biomarkers.

### Literature Search Methodology

To conduct this comprehensive review, a systematic literature search strategy was employed across the PubMed/MEDLINE and Scopus databases. Search strings included combinations of (“uterine leiomyosarcoma” OR “uLMS” OR “STUMP” OR “leiomyoma”) AND (“oxidative stress” OR “galectin-1” OR “microRNA” OR “biomarkers” OR “tumor microenvironment”). Inclusion criteria were limited to peer-reviewed articles published in English, primarily fousing on the last 15 years to capture contemporary molecular data. Studies were selected based on their relevance to the molecular pathogenesis, epigenetic mechanisms, and diagnostic biomarkers of uterine smooth muscle tumors. Both human clinical cohorts and experimental in vitro models were included, provided they explicitly contributed to the understanding of the targeted pathways.

## 2. Anatomy and Physiological Microenvironment

### 2.1. Uterine Anatomy and the Myometrial Structure

The uterus is a hollow, muscular organ centrally located in the lesser pelvis, situated precisely between the urinary bladder anteriorly and the rectum posteriorly. Anatomically, it is divided into three primary sections: the body (corpus), the isthmus, and the cervix. The uterine cavity maintains a triangular shape within the corpus and directly communicates with the fallopian tubes via the bilateral tubal ostia. The organ is securely maintained in its anatomical position by a highly complex supportive mechanism consisting of the broad ligaments, uterosacral ligaments, transverse ligaments, and round ligaments. These ligamentous structures work in essential conjunction with the pelvic floor muscles and fascia to support the pelvic organs [8].

The uterine wall consists of three distinct microscopic layers:

Endometrium: The inner mucosal layer that undergoes profound cyclical changes during the menstrual cycle. It is functionally divided into a basal layer and a functional layer, the latter of which is shed during menstruation and regenerates sequentially from the basal layer.
Myometrium: The thickest layer of the uterine wall, strictly composed of smooth muscle fibers. These fibers are organized in diverse and overlapping orientations, including longitudinal, circular, and oblique bundles. This intricate structural organization allows for the generation of strong, coordinated contractions necessary for endometrial shedding, the maintenance of pregnancy, and parturition.
Perimetrium: The outer serous layer, representing a direct extension of the peritoneum that covers the majority of the uterine surface.

The vascular supply to this highly metabolically active tissue is primarily provided by the uterine arteries, which are direct branches of the internal iliac arteries. These arteries branch extensively within the myometrium to form specialized arcuate and radial arteries that supply the underlying endometrium. Innervation is derived from the autonomic nervous system via the hypogastric plexus, encompassing both sympathetic and parasympathetic fibers that meticulously regulate muscular and vascular function [8].

### 2.2. Hormonal Regulation and Dysregulation

The functional integrity of the myometrium is heavily regulated by complex hormonal, neural, and local signaling networks. Estrogens stimulate the rapid proliferation of myometrial cells, while progesterone primarily drives cellular differentiation and functional maturation. The disruption of these signaling pathways, compounded by oxidative cellular stress, correlates with abnormal cellular proliferation and the excessive deposition of extracellular matrix. This specific hormonal and oxidative interplay participates in the formation and sustained growth of uterine leiomyomas [9].

## 3. Histopathological and Clinical Classifications

The World Health Organization (WHO) and the International Federation of Gynecology and Obstetrics (FIGO) provide the standard frameworks for classifying these tumors, relying heavily on morphological criteria and anatomical positioning [10].

### 3.1. Benign Tumors: Leiomyoma Subtypes

Leiomyomas are characterized by well-differentiated smooth muscle cells, minimal cytologic atypia, low mitotic indices, and a complete absence of tumor cell necrosis. The WHO recognizes several morphological subtypes that hold particular diagnostic significance:

- Classic (usual) leiomyoma.
- Cellular leiomyoma, which features dense cellularity but lacks malignant criteria.
- Atypical or bizarre leiomyoma, which may mimic malignancy due to nuclear pleomorphism.
- Mitotically active leiomyoma, showing increased mitotic counts but lacking atypia or necrosis.
- Epithelioid leiomyoma.
- Myxoid leiomyoma [10].

Furthermore, the FIGO PALM-COEIN system classifies leiomyomas anatomically to guide surgical intervention:

Type 0: Pedunculated intracavitary leiomyoma without intramural extension.
Type 1: Submucosal leiomyoma with less than 50% intramural extension.
Type 2: Submucosal leiomyoma with 50% or more intramural extension.
Type 3: Intramural leiomyoma in direct contact with the endometrium, not protruding into the cavity.
Type 4: Purely intramural leiomyoma.
Type 5: Subserosal leiomyoma with 50% or more intramural involvement.
Type 6: Subserosal leiomyoma with less than 50% intramural involvement.
Type 7: Pedunculated subserosal leiomyoma.
Type 8: Leiomyomas of other specified locations (e.g., cervical, intraligamentary) [11].

### 3.2. Smooth Muscle Tumors of Uncertain Malignant Potential (STUMPs)

STUMPs constitute a diagnostically challenging entity featuring atypical morphology that does not completely satisfy the criteria for outright malignancy [12]. Diagnosis relies strictly on evaluating the degree of cellular atypia, the exact mitotic activity, and the presence or absence of tumor cell necrosis. Their clinical behavior is notoriously unpredictable. While most STUMPs follow a relatively benign course, they carry a documented recurrence rate ranging from 8.7% to 11% [12]. Recurrences may present as recurrent STUMP or, more dangerously, undergo malignant transformation into fully aggressive leiomyosarcomas [6]. It is critical to note that STUMP and uLMS do not necessarily exist on a simple biological continuum. STUMPs should not automatically be considered a direct presursor lesion of uLMS; they are distinct clinical entities with variable biological trajectories.

### 3.3. Malignant Counterparts: uLMS and Associated Sarcomas

Malignant neoplasms of the myometrium primarily include leiomyosarcomas, endometrial stromal sarcomas (ESSs), and undifferentiated uterine sarcomas:

Uterine Leiomyosarcoma (uLMS): Diagnosis depends rigidly on the Stanford criteria within the context of the WHO classification. These criteria require the presence of at least two of the following morphological features: diffuse and severe cytologic atypia, a high mitotic index (typically greater than 10 mitoses per 10 high-power fields), and coagulative tumor cell necrosis [12]. Nevertheless, the diagnosis cannot be reduced to a simple “2 out of 3” checklist. Histological variants, such as myxoid leiomyosarcoma, may exhibit lower apparent mitotic activity, drastically complicating the histopathological diagnosis and requiring nuanced evaluation. uLMSs typically express definitive smooth muscle markers such as desmin, smooth muscle actin (SMA), and h-caldesmon. They frequently demonstrate a marked loss of estrogen and progesterone receptor (ER and PR) expression, rendering hormonal therapies ineffective [13].
Endometrial Stromal Sarcoma (ESS): Differentiated into low-grade and high-grade malignancies, originating from the endometrial stroma. Low-grade ESS often expresses hormonal receptors and exhibits an indolent but recurrent course, whereas high-grade ESS is highly aggressive.
Undifferentiated Uterine Sarcoma: A rare, extraordinarily aggressive neoplasm characterized by a complete absence of histological differentiation, severe atypia, and extensive necrosis.

## 4. Oxidative Stress: Associative Mechanisms and Genomic Instability

The progression from a normal myometrial cell to a highly aggressive sarcoma is deeply underpinned by a profound deterioration of genomic integrity. While oxidative stress is strongly associated with this deterioration, it is vital to distinguish between oxidative stress as a marker of cellular burden versus a definitive, initiating cause of uLMS.

### 4.1. Markers of Oxidative Burden in Uterine Tumors

The tumor microenvironment in uLMS and rapidly growing leiomyomas is characterized by a severe imbalance in oxidative homeostasis.

Oxidative stress is defined as the chronic disruption of the equilibrium between reactive oxygen species (ROS) production and the body’s endogenous antioxidant defenses [14]. Clinical studies have revealed a significant increase in systemic and local protein oxidative stress status in women harboring uterine leiomyomas [14] (Table 1). Specific markers of oxidative stress, notably lipid peroxidation products such as malondialdehyde (MDA), have been extensively studied and are highly elevated in leiomyosarcomas. However, an elevated MDA may indicate increased lipid peroxidation but does not independently prove that MDA is a specific diagnostic marker of uLMS or a primary factor causing malignant transformation. Concentrations of serum protein carbonyl groups and advanced oxidized protein products (AOPPs) are significantly higher in clinical cohorts of leiomyoma patients compared to healthy controls [14]. By contrast, serum thiol levels, which reflect critical antioxidant capacity, are markedly lower in patients harboring uterine tumors [14].

Leiomyoma cells frequently exhibit lower expression of antioxidant enzymes, such as superoxide dismutase (SOD) and catalase (CAT), compared to normal uterine muscle cells, specifically in hypoxic environments.

Transcriptome-combined Mendelian randomization analyses have identified highly specific oxidative stress-related biomarkers in uterine leiomyoma, establishing genes such as *ANXA1*, *CD36*, *MICB*, and *PRDX6* as potential mediators in tumor risk stratification [15].

### 4.2. Metabolic Reprogramming and Hypoxia

The profound oxidative stress observed in uLMS is closely tied to the metabolic reprogramming of the tumor, specifically the metabolic shift toward anaerobic glycolysis known as the Warburg effect [16]. Lactate dehydrogenase (LDH) is a key enzyme in this process, converting pyruvate to lactic acid under anaerobic conditions [16]. LDH consists of five distinct isoenzymes (LDH-1 through LDH-5). LDH-1 is prevalent in cardiac tissue; LDH-2 in the reticuloendothelial system; LDH-3 in the lungs; LDH-4 in the kidneys; and LDH-5 predominantly in the liver and skeletal muscle [17]. In sarcomas of smooth muscle origin, there is a characteristic, pathological elevation in the LDH-4 and LDH-5 isoenzymes. The predominance of the LDH-5 isoenzyme is heavily associated with severely hypoxic regions within the tumor microenvironment [17] (Table 1). The Magna Graecia score attempts to utilize this metabolic shift by combining serum LDH levels with MRI characteristics, tumor growth rates, and menopausal status to improve preoperative diagnosis. However, this score and the diagnostic utility of LDH-5 must be interpreted extremey cautiously. The studies evaluating this score are based on relatively small, localized clinical cohorts. Without large-scale prospective validation establishing exact sensitivity, apecificity, and uniform methodologies (such as distinguishing whether total LDH or specifically LDH-5 was measured in clinical assays), LDH-5 cannot yet be presented as a clincially validated tool for uLMS diagnosis [17].

### 4.3. Mutational Landscape and Molecular Heterogeneity

The severe genomic instability observed in uLMS manifests as a distinct and highly volatile mutational landscape. It is critical to emphasize that uLMS is molecularly heterogenous, and there is no single simple “molecular pathway” responsible for all cases.

*TP53*, a critical tumor suppressor gene responsible for cell cycle regulation and DNA repair, is mutated or deleted in 60% to 80% of all uLMS cases [18]. Inactivation of the *RB1* gene occurs in approximately 50% of these tumors, further dismantling essential cell cycle checkpoints [18].

Alterations in the *ATRX* gene are found in 25% to 35% of uLMS cases and are intrinsically linked to the alternative lengthening of telomeres (ALT). This ALT mechanism bypasses standard cellular senescence, conferring replicative immortality to the cancer cells [19].

It is vital to distinguish alterations characteristic of benign leiomyomas, such as *MED12* mutations, from those associated with uLMS. MED12 is a typical hallmark of benign disease and should not be misconstructed as a driving alteration in uLMS, further underscoring the divergence between benign and malignant molecular pathways.

Cytogenetically, uLMS reveals complex, highly aneuploid karyotypes featuring frequent chromosomal additions at 1q and 17p, and critical deletions at chromosome 10q (the PTEN locus) and 13q14 (the RB1 locus) [19].

## 5. Epigenetic Dysregulation in the Stressed Microenvironment

The genomic instability and oxidative stress inherent to uLMS create a highly dysregulated epigenetic landscape, primarily characterized by aberrant microRNA (miRNA) expression.

### 5.1. miRNA Biogenesis and Extracellular Stability

miRNAs are small, non-coding RNAs comprising approximately 18 to 25 nucleotides that modulate gene expression post-transcriptionally [20]. The biogenesis of miRNAs begins in the nucleus with the transcription of primary miRNAs (pri-miRNAs) by RNA polymerase II. These are processed by the Drosha-DGCR8 microprocessor complex into 70-nucleotide precursor miRNAs (pre-miRNAs). Pre-miRNAs are exported to the cytoplasm via Exportin-5, where they undergo enzymatic cleavage by Dicer to produce mature double-stranded miRNA duplexes [20,21]. The guide strand is incorporated into the RNA-induced Silencing Complex (RISC) to direct target mRNA recognition, while the passenger strand is typically degraded.

miRNAs are extraordinarily stable in the extracellular environment. They circulate securely protected from endogenous RNases by associating with proteins such as Argonaute 2 (Ago2) or high-density lipoproteins (HDLs), or by encapsulation within extracellular vesicles (EVs) and exosomes. This structural protection allows miRNAs to withstand harsh physical conditions, making them ideal targets for non-invasive liquid biopsies detected via RT-qPCR, microarray, next-generation sequencing (NGS), and digital droplet PCR (ddPCR) [21].

### 5.2. Downregulation of Tumor-Suppressive miRNAs

The tumor microenvironment is associated with the silencing of miRNAs that normally act as tumor suppressors, thereby correlating with cellular proliferation and invasion (Table 2).

Expression of the miR-29 family is significantly downregulated in uLMS tissue samples. miR-29b is responsible for targeting essential oncogenes, including *AKT3* and *CDK6*, as well as extracellular matrix (ECM) remodeling genes. The loss of miR-29 expression is associated with the hyperactivation of oncogenic signaling cascades and is robustly associated with poor patient prognosis [22].

Oxidative stress and hypoxia are known factors associated with epithelial–mesenchymal transition (EMT). The miR-200 family normally acts to inhibit EMT by directly targeting the potent transcription factor *ZEB1* in in vitro models [23]. The downregulation of the miR-200 family in uLMS tissues is implicated in the acquisition of an invasive, mesenchymal phenotype.

The let-7 Family of miRNAs suppresses multiple highly active oncogenes, including *HMGA2*, *RAS*, and *MYC* [24]. The downregulation of let-7 in uLMS removes crucial growth suppression mechanisms.

Additional Suppressors, such as miR-34a and miR-181b, specifically function to inhibit invasion and cellular migration in in vitro models of uterine mesenchymal tumors; their loss is associated with metastatic potential. Similarly, the downregulation of miR-10b-5p enhances proliferation and correlates with a highly aggressive phenotype. Downregulation of miR-126 removes the suppression of *VEGF*, leading to uninhibited angiogenesis.

### 5.3. Upregulation of Oncogenic miRNAs (OncomiRs)

Conversely, the microenvironment exhibits overexpression of specific miRNAs that are implicated in tumor survival (Table 2).

One of the most consistently overexpressed miRNAs in uLMS tissue is miR-21. It functions as an oncomiR by targeting and inhibiting critical tumor suppressors such as PTEN and PDCD4 in experimental models [25]. The concurrent upregulation of miR-21 and the downregulation of miR-29 is associated with synergistic hyperactivation of the PI3K/AKT/mTOR signaling pathway.

The miR-183~96~182 cluster is highly upregulated in uLMS. Specifically, in vitro studies indicate that miR-182 targets tumor suppressors like FOXO1, enhancing the migratory capacity of the cells [26].

Overexpression of the miR-221/222 cluster is implicated in cell cycle dysregulation and is associated with chemotherapy resistance across various sarcomas in tissue-based studies [27].

## 6. Glycoprotein and Molecular Biomarkers: Critical Assessment

The oxidative and hypoxic conditions within the uLMS microenvironment correlate with the expression of surface and circulating glycoproteins, which may participate in angiogenesis and immune evasion [28]. It is crucial to strictly separate established diagnostic biomarkers from experimental mechanistic mediators.

### 6.1. Galectin-1

Galectin-1 is a β-galactoside-binding lectin that may play a role in the tumor’s adaptation to stress.

Pathophysiology: Galectin-1 is overexpressed in various neoplasms. Within the tumor microenvironment, it is associated with enhanced angiogenesis via VEGF-dependent mechanisms. Furthermore, in vitro and animal models suggest it creates an immunosuppressive environment by inducing apoptosis in activated T-lymphocytes [28].

Clinical Diagnostic Performance: A prospective case–control study analyzed serum samples from 156 women, comprising 49 healthy controls, 90 patients with benign uterine myomas, and 17 patients with aggressive uterine sarcomas or STUMPs (Table 1). The study found that serum galectin-1 concentrations are significantly higher in patients with uterine sarcomas and STUMPs compared to both healthy controls and patients with benign leiomyomas (*p* = 0.002) [29]. In stringent multivariable logistic regression analyses, adjusted for critical clinical variables such as patient age and menopausal status, galectin-1 remained independently and strongly associated with the presence of sarcoma (OR 1.07 per 1000-unit increase; 95% CI 1.02–1.13; *p* = 0.007) [29]. Receiver operating characteristic (ROC) curve analysis revealed a moderate discriminative ability for galectin-1, yielding an Area Under the Curve (AUC) of 0.78 when distinguishing sarcomas from healthy controls, and an AUC of 0.72 when comparing sarcomas to all non-sarcoma cases combined.

Limitations: At its optimal calculated cutoff value, galectin-1 achieved high specificity (100%) in the investigated cohort [28]. However, this finding must be interpreted with extreme caution. The study cohort included only 17 sarcoma/STUMP patients, and the sensitivity was severely limited at 47%. These results do not justify the conclusion that galectin-1 is an independent biomarker capable of reliably diagnosing uLMS in broad clinical practice. Larger, multicenter, and independently validated studies are strictly required before clinical application.

### 6.2. Mucin-1 (MUC1) and Inflammatory Markers

MUC1 is a highly glycosylated, high-molecular-weight transmembrane protein. In many epithelial malignancies, MUC1 is aberrantly glycosylated and overexpressed [30]. Despite its vital role in epithelial cancers, MUC1 lacks diagnostic utility in uterine mesenchymal tumors [31]. The aforementioned clinical study evaluated circulating serum MUC1 levels across the 156-patient cohort and found no statistically significant differences between healthy controls, myoma patients, and sarcoma patients (*p* = 0.178) [29] (Table 1).

Elevated C-Reactive Protein (CRP) in uLMS patients correlates with an inflammatory microenvironment but lacks diagnostic specificity, as it rises in numerous benign conditions. Similarly, while the overexpression of pro-inflammatory cytokines like IL-6 and IL-8 is observed, their clinical diagnostic use remains strictly experimental.

## 7. Clinical Translation and Future Directions

### 7.1. Enhancing Preoperative Diagnostics and Imaging

Currently, transvaginal ultrasound and MRI serve as the primary clinical imaging modalities, but they cannot definitively differentiate benign from malignant disease [32].

The MYLUNAR (MYometrial Lesion UltrasouNd And mRi) study attempts to utilize specific ultrasound parameters (e.g., highly irregular margins, internal cystic degeneration, and elevated vascular Doppler parameters like Resistance Index and Pulsatility Index) alongside MRI functional indicators, specifically Diffusion-Weighted Imaging (DWI) and the Apparent Diffusion Coefficient (ADC), to flag suspicious masses.

While circulating miRNAs, such as elevated serum miR-1246 and miR-191-5p, show promise in initial clinical cohorts, and targeted tissue-based panels (miR-144-3p, miR-34a-5p, miR-206) have been identified, moving from experimental biomarkers to clinical application requires rigorous scrutiny [33]. The sequence of translating a biomarker into reliable liquid biopsy for preoperative diagnosis and risk stratification has not yet been clinically established for uLMS.

When evaluating these proposed diagnostic panels, significant demographic and clinical confounders must be accounted for. Oxidative stress markers and glycoprotein levels can be heavily skewed by a patient’s age, body mass index (BMI), menopausal status, and parity. Furthermore, a benign leiomyoma undergoing natural ischemic degeneration can produce immense localized inflammation and elevate total LDH and oxidative stress markers, severely confounding biomarker panels attempting to diagnose uLMS [34].

### 7.2. Surgical Management and the Morcellation Controversy

The absence of reliable preoperative diagnostics directly impacts surgical safety. Because aggressive uLMS is frequently diagnosed incidentally following minimally invasive surgery for presumed benign fibroids, the use of uncontained laparoscopic power morcellation carries a documented risk of rapid tumor upstaging and intraperitoneal dissemination. In response to these severe risks, the FDA strongly recommends against the use of power morcellation in the majority of women, mandating containment systems only in carefully selected, definitively low-risk patients [7].

Biomarkers like galectin-1 and specific miRNA profiles may have the potential to improve preoperative risk stratification. However, prospective validation reporting robust sensitivity, specificity, positive predictive value (PPV), and negative predictive value (NPV) in large, independent cohorts is absolutely required before they can be clinically implemented to guide surgical decision-making.

### 7.3. Experimentl Therapeutics

For advanced or recurrent uLMS, systemic chemotherapy regimens remain the standard of care, yet they offer only modest progression-free survival. Understanding the molecular pathways associated with oxidative stress has led to novel experimental avenues, though these remain largely preclinical. In vitro preclinical research demonstrates that inducing fatal levels of oxidative stress via proteasome inhibition using bortezomib impacts uLMS cell lines (e.g., SK-LMS-1 and SK-UT-1B). In these cell line models, bortezomib treatment induces massive reactive oxygen species production and profound mitochondrial membrane depolarization [35]. Animal studies and preclinical models show that the PI3K/mTOR pathway is frequently hyperactivated. Preclinical evidence suggests that inhibiting the PI3K/mTOR pathway physically remodels the tumor microenvironment and successfully sensitizes the sarcoma to PD-1 immune checkpoint blockade in vivo [36]. Additionally, miRNA-based therapeutics remain strictly experimental. In vitro models investigating synthetic miRNA mimics aim to artificially replenish lost tumor suppressors (e.g., miR-34a mimics) [37] and specialized antagomiRs designed to silence oncogenic drivers like miR-221/222 show mechanistic potential, but their clinical efficacy and safety profiles are entirely unproven in human uLMS.

## 8. Conclusions

Uterine leiomyosarcoma is an aggressive, heterogeneous malignancy characterized by profound genomic instability. The tumor microenvironment is deeply marked by altered antioxidant defenses, elevated lipid peroxidation (MDA), and metabolic reprogramming, all of which are strongly associated with the disease’s progression. This microenvironment exhibits widespread epigenetic dysregulation, specifically the suppression of tumor-protective miRNA families (miR-29, miR-200) and the overexpression of oncogenic miRNAs (miR-21, miR-183~96~182).

Simultaneously, this microenvironment promotes the expression of galectin-1. While galectin-1 demonstrates high specificity in distinguishing uLMS from benign leiomyomas in early cohorts, its low sensitivity and the limited sample sizes of current studies mean it cannot yet be utilized as a reliable, independent diagnostic tool.

To definitively overcome the diagnostic “gray zone” that currently plagues the surgical management of uterine smooth muscle tumors, future oncological research must prioritize the rigorous standardization of oxidative stress assays. Most importantly, any proposed biomarker panels integrating miRNA signatures and galectin-1 quantification require systemic, prospective validation in appropriately powered, independent clinical cohorts before they can safely be introduced for preoperative risk stratification and surgical management.

## Figures and Tables

**Table 1:** Summary of biomarker studies in uterine smooth muscle tumors.

Biomarker	Biological Material	Study Type	Findings in uLMS/STUMP	Diagnostic Value	Main Limitation/Level of Evidence
**Galectin-1**	Serum	Clinical Cohort	Significantly elevated vs. leiomyomas and controls.	AUC 0.78. High specificity (100%), low sensitivity (47%).	Small sample size (*n* = 17 uLMS/STUMP). Requires independent validation.
**MUC1**	Serum	Clinical Cohort	No significant difference across groups.	AUC 0.59. Poor discriminative ability.	Not a viable independent biomarker for uLMS.
**LDH-5**	Serum/Tissue	Clinical Cohort	Elevated; predominates in hypoxic tumor regions.	Incorporated into Magna Graecia score.	Lack of large prospective cohorts; score lacks independent validation.
**MDA**	Serum	Clinical Cohort	Elevated compared to healthy controls.	Indicator of oxidative stress/lipid peroxidation.	Not a specific diagnostic biomarker. Associative finding.

**Table 2:** Key epigenetic signatures in uterine leiomyosarcoma.

miRNA Signature	Expression Profile	Validated Target Genes	Biological Consequence	Level of Evidence
miR-29 Family	Downregulated	*AKT3*, *CDK6*, ECM genes	Associated with PI3K pathway hyperactivation	Human Tissue/Clinical Cohorts
miR-200 Family	Downregulated	*ZEB1*	Implicated in loss of EMT suppression	In Vitro/Cell Lines
let-7 Family	Downregulated	*HMGA2*, *RAS*, *MYC*	Associated with uncontrolled cell cycle	Human Tissue
miR-21	Upregulated	*PTEN*, *PDCD4*	OncomiR activity; hyperactivation of PI3K/AKT/mTOR	In Vitro/Human Tissue
miR-183~96~182	Upregulated	*FOXO1*	Correlates with enhanced migratory capacity	Human Tissue/In Vitro
miR-221/222	Upregulated	Cell cycle regulators	Associated with promotion of proliferation	Human Tissue

## Data Availability

No new data were created or analyzed in this study. Data sharing is not applicable to this article.

## Abbreviations

The following abbreviations are used in this manuscript:

uLMS	Uterine Leiomyosarcoma
STUMP	Smooth Muscle Tumor of Uncertain Malignant Potential
miRNA	microRNA
MDA	Malondialdehyde
MUC1	Mucin-1
MRI	Magnetic Resonance Imaging
ROS	Reactive Oxygen Species
WHO	World Health Organization
FIGO	International Federation of Gynecology and Obstetrics
ESS	Endometrial stromal sarcomas
SMA	Smooth muscle actin
ER/PR	Estrogen Receptor/Progesterone Receptor
AOPP	Advanced Oxidized Protein Products
SOD	Superoxide dismutase
CAT	Catalase
LDH	Lactate Dehydrogenase
ALT	Alternative Lengthening of Telomeres
Pri-miRNA	Primary miRNA
Pre-miRNA	Precursor miRNA
RISC	RNA-induced Silencing Complex
Ago2	Argonaute 2
HDL	High-density lipoproteins
EV	Extracellular vesicles
NGS	Next-generation sequencing
ddPCR	Digital droplet PCR
ECM	Extracellular matrix
EMT	Epithelial–Mesenchymal Transition
oncomiRs	Oncogenic miRNAs
ROC	Receiver operating characteristic
AUC	Area Under the Curve
CRP	C-Reactive Protein
MYLUNAR	MYometrial Lesion UltrasouNd And mRi
DWI	Diffusion-Weighted Imaging
ADC	Apparent Diffusion Coefficient
BMI	Body mass index
PPV	Positive predictive value
NPV	Negative predictive value
